# Selective metal passivation by vapor-dosed phosphonic acid inhibitors for area-selective atomic layer deposition of SiO_2_ thin films

**DOI:** 10.1186/s40580-025-00490-5

**Published:** 2025-05-30

**Authors:** Jeong-Min Lee, Seo-Hyun Lee, Ji Hun Lee, Junghun Kwak, Jinhee Lee, Woo-Hee Kim

**Affiliations:** 1 Department of Materials Science and Chemical Engineering, BK21 FOUR ERICA-ACE Center, Hanyang University, Ansan, Gyeonggi 15588 Republic of Korea; 2 SK Specialty Co., Ltd, 59-33 Gaheunggongdan-ro, Yeongju-Si, Gyeongsangbuk-Do 36059 Korea

**Keywords:** Area-selective atomic layer deposition, Silicon oxide, Phosphonic acid, Chemical vapor transport, Selective removal

## Abstract

Aiming for atomic-scale precision alignment for advanced semiconductor devices, area-selective atomic layer deposition (AS-ALD) has garnered substantial attention because of its bottom-up nature that allows precise control of material deposition exclusively on desired areas. In this study, we develop a surface treatment to hinder the adsorption of Si precursor on metal surfaces by using a vapor-phase functionalization of bulky phosphonic acid (PA) self-assembled monolayers (SAMs). Through the chemical vapor transport (CVT) method, the bulky solid PA inhibitor with a fluorocarbon terminal group was effectively vaporized, and the conditions for maximizing the blocking effect of the inhibitor were confirmed by optimizing the process temperature and dwelling time. The unintended PA inhibitors adsorbed on SiO_2_ surfaces during the CVT process were selectively removed by post-HF treatment, thereby leading to selective deposition of SiO_2_ thin films only on SiO_2_ substrates. As a results, SiO_2_ film growth on the PA SAM/HF-treated TiN surfaces was suppressed by up to 4 nm with just a single exposure to the long-chain inhibitor, even during the ALD process using highly reactive O_3_ reactants. The proposed approach paves the way for highly selective deposition of dielectrics on dielectrics (DoD).

**Graphical Abstract:**

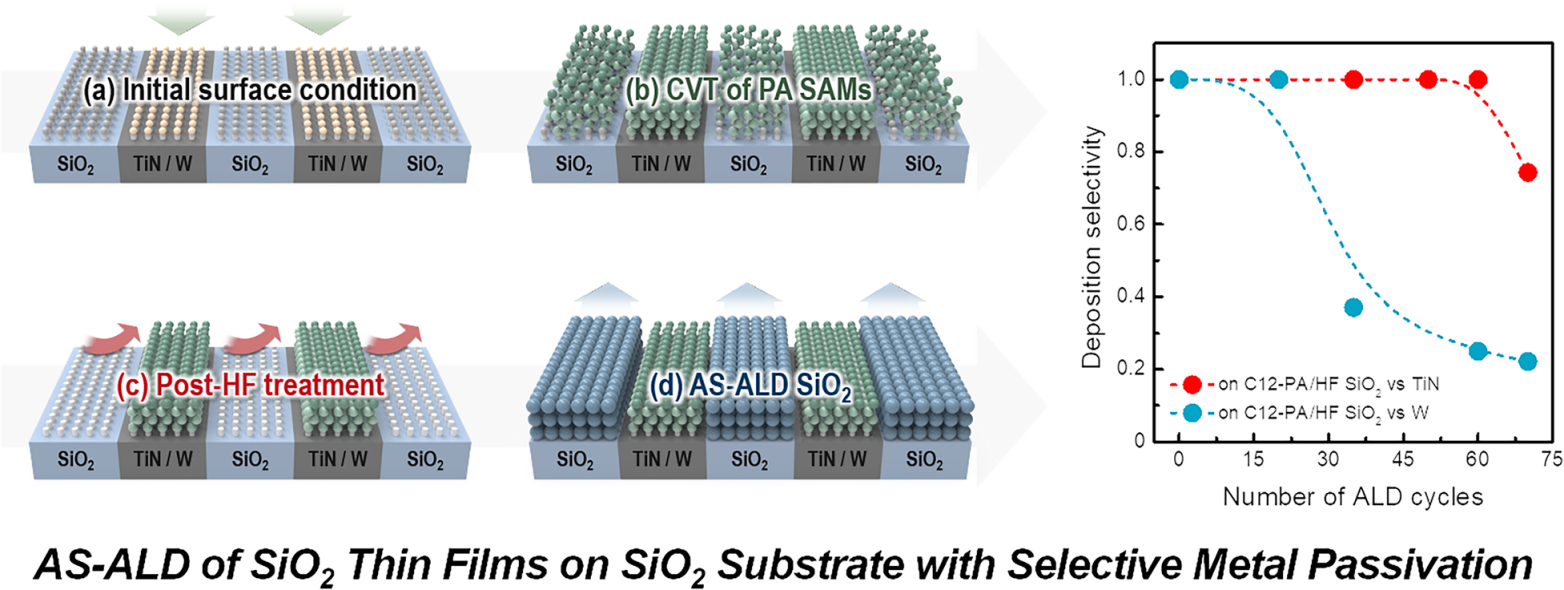

**Supplementary Information:**

The online version contains supplementary material available at 10.1186/s40580-025-00490-5.

## Introduction

As devices shrink and patterning alignment requires atomic-level accuracy, area-selective atomic layer deposition (AS-ALD) is currently gaining traction in the semiconductor industry [1–3]. AS-ALD ensures high-precision patterning due to its self-aligned and surface self-limiting nature, which allows selective material growth only on desired areas (referred to as the ‘growth area’) by exploiting the chemical differences between different underlying substrates. This feature of AS-ALD not only eliminates misalignment issues (i.e., edge-placement error), but also simplifies the fabrication steps compared to conventional lithography-based top-down patterning processes [4–6]. The performance of the AS-ALD is typically described in terms of selectivity, which refers to the amount of material deposited on the growth area relative to the non-growth area, where film growth is not desired, and it can be further improved by controlling surface chemical reactivity [7, 8].

Although several studies have achieved AS-ALD by utilizing inherent chemical reactivity differences without additional surface treatment, [9–12] a more general and potentially applicable solution for blocking film deposition across various material systems is to use surface passivation. So far, versatile surface manipulation techniques, such as plasma treatment, [13, 14] ion implantation, [15, 16] polymers [17, 18], self-assembled monolayers (SAMs), [19–22] and small molecule inhibitors (SMIs), [23–25] have been employed to deactivate non-growth regions. In this approach, surface functional groups are modified with hydrogen (-H), methyl (-CH_3_), and trifluoromethyl (-CF_3_) moieties, which block and hinder the adsorption of precursor molecules, allowing selective film deposition on growth regions. These modifications can be performed either before or during the ALD process to maximize the adsorption selectivity of precursors between growth and non-growth areas. Note that recent studies have extensively reported the insertion of SMIs during the ALD process to enhance deposition selectivity, as their high vapor pressure facilitates gas-phase processing [26–28].

However, SMIs have structural limitations compared to SAM molecules. SMIs can be described similarly to SAMs in that they consist of a reactive moiety that binds to the substrate and an inert group that blocks precursor adsorption, but the intermolecular forces between the adsorbed SMIs are too weak to induce surface alignment, resulting in a disordered inhibitor layer with low coverage [24, 29]. In contrast, the self-assembly characteristics of SAMs are driven by van der Waals interactions between adjacent large aliphatic chains, which contribute to the formation of a well-ordered and densely packed inhibition layer, leading to significantly longer nucleation delays in the subsequent ALD process [24, 30]. Considering that the inhibitory properties of these SAMs are proportional to chain length, we anticipated that the vapor application of long-chain SAMs would offer advantages in blocking performance, process time, and compatibility with the ALD process. Indeed, in our recent work that uses vapor dosing and selective removal of octadecylphosphonic acid (ODPA), we successfully demonstrated 12 nm of selective growth of ZrO_2_ thin films on SiO_2_ versus TiN and W substrates [31]. By using chemical vapor transport (CVT) process that can effectively vaporize solid inhibitors, ODPA SAMs were formed in just 10 min compared to the conventional solution-based coating process that required more than 24 h. It should be noted that the ODPA layer produced by the CVT process exhibited excellent blocking properties when O_2_ was used as a reactant in subsequent ALD processes. However, rapid degradation occurred under exposure to strong oxidizing agents such as ozone (O_3_). This ongoing challenge in overcoming the limitation of reactant choice prompted our investigations, and the aim of this study is therefore to further expand the AS-ALD process to other systems using co-reactants with higher reactivity.

In the present study, we report an AS-ALD approach based on surface modification utilizing fluorine-containing phosphonic acid SAMs formed via the CVT process on SiO_2_, TiN, and W substrates to attain dielectric versus metal selectivity. For broader applicability, the AS-ALD process, which selectively grows SiO_2_ thin films only on SiO_2_ substrate, was used as a model system to verify the surface modification. Since the Si precursor has low reactivity toward the surface or reactants, an ALD process using aggressive oxygen sources, such as O_3_ or O_2_ plasma, is generally required to deposit SiO_2_ thin films at low temperatures where inhibitor degradation does not occur [32, 33]. Herein, a low temperature ALD SiO_2_ capable of producing high-quality SiO_2_ thin films at deposition temperatures as low as 50 ℃, using the 1,2-bis(diisopropylamino) disilane (BDIPADS) precursor and O_3_ as the reactant, was employed as the primary process for subsequent evaluation [34, 35]. We first adjusted the CVT process variables, including process temperature, dwell time, and sample position, to determine optimal conditions for forming a well-aligned inhibitor layer, and the performance of fluorine-containing SAMs was then evaluated through subsequent ALD SiO_2_ processes. Selective deposition of SiO_2_ thin films on dielectric versus metal substrates was achieved by introducing a selective removal process to restore the SiO_2_ surfaces, allowing for selective deposition of 4.5 nm-thick SiO_2_ thin films on SiO_2_ substrates compared to TiN substrates at low temperatures of 100 ℃. Our findings suggest that bulky SAMs with long chain lengths and high molecular weights exceeding 600 g/mol can effectively be applied in the vapor-phase processes, enabling the selective deposition of SiO_2_ films on SiO_2_ substrates, even under exposure to strong oxidizing O_3_ reactants.

## Experimental methods

Experiments were performed on various substrates including blanket SiO_2_ (18 nm)/Si, TiN (15 nm)/SiO_2_/Si, and W (100 nm)/TiN/SiO_2_/Si substrates. Phosphonic acid (PA) SAMs were used to confer oxide versus metal selectivity against subsequent SiO_2_ ALD process, and each substrate surface was modified through both solution and vapor-phase treatment of PA SAMs. For solution SAM treatment, (3,3,4,4,5,5,6,6,7,7,8,8,9,9,10,10,11,11,12,12,12-Heniscosafluorododecyl) phosphonic acid (denoted as ‘C12-PA’, Sigma-Aldrich), (3,3,4,4,5,5,6,6,7,7,8,8,9,9,10,10,10-Heptadecafluorodecyl) phosphonic acid (denoted as ‘C10-PA’, Sigma-Aldrich), and (3,3,4,4,5,5,6,6,7,7,8,8,8-Tridecafluorooctyl) phosphonic acid (denoted as ‘C8-PA’, Sigma-Aldrich) were used. After cleaning, the substrates were immersed in a 1 mM solution of the PA SAMs in toluene solvents held at a room temperature for 24 h. For vapor SAM treatment, a chemical vapor transport (CVT) process was employed to effectively vaporize the solid C12-PA SAMs using a customized 4 channel inhibitor coater (Moman Co.) controlled by LabVIEW software. To minimize the influence of the temperature gradients within the quartz chamber, the samples were placed at the center of the chamber, which served as the reference point for all temperature settings. The chamber was then pumped down to a base pressure of ~ 0.05 Torr. Following a short pre-N_2_ purge of 1 min at a working pressure of ~ 1 Torr, the valve connected to the rotary pump was close to allow a defined residence time for the inhibitor exposure. The C12-PA SAMs were treated onto substrates at a controlled temperature of 50 to 300 ℃ for 1 min to 3 h, followed by a 5 min of N_2_ purging step. After vapor dosing of SAMs, the samples were cleaned in diluted hydrogen fluoride solution (HF, 10 wt.%) for 1 s to strip away PA SAMs adsorbed on the SiO_2_ surfaces. The HF treatment was sufficiently short to avoid etching the underlying SiO_2_ substrates. Following the HF treatment, the samples were immediately transferred to the ALD reactor for subsequent processing.

Then, SiO_2_ films were deposited on various substrates using an atomic layer deposition technique in a traveling-wave type reactor (Atomic classic, CN-1). The deposition process involved the use of 1,2-bis(diisopropylamino)disilane (BDIPADS) and O_3_ as the precursor and counter reactant, respectively. The BDIPADS precursor was vaporized in a bubbler-type canister at room temperature, with delivery lines heated to 80 ℃ to prevent the condensation of the precursor. High-purity N_2_ (99.999%) was employed as a carrier gas at a flow rate of 300 sccm to remove excess gas molecules and byproducts generated during the surface reaction. The SiO_2_ ALD sequence included the following steps: 2 s of BDIPADS exposure, 30 s of precursor purge, 5 s of O_3_ exposure, and 60 s of reactant purge. All SiO_2_ ALD processes were performed at a low deposition temperature of 100–150 ℃ to minimize deterioration of the inhibitor molecules. Further detailed information on the SiO_2_ ALD process for BDIPADS precursors using O_3_ reactant can be found in our earlier publication [35].

The surface hydrophobicity was estimated at three different spots via static water contact angle (WCA) measurements using a contact angle analyzer (SDL200TEZD, FEMTOFAB), and the average value was taken to represent the hydrophobicity of each surface after inhibitor coating. Film thickness was measured using a spectroscopic ellipsometer (MG-1000, Nano View Co.) with a spectral range of 380–900 nm and field emission transmission electron microscopy (FE-TEM, JEM-2100F HR, JEOL Ltd.). The element composition and chemical binding structures of various surfaces was examined via X-ray photoelectron spectroscopy (XPS) (K-alpha plus, Thermo Fisher Scientific Co.) using Al Kα as the X-ray source, where the C–C peak at 284.8 eV was used as a reference to calibrate the measured core levels.

## Results and discussion

**Fig. 1:**
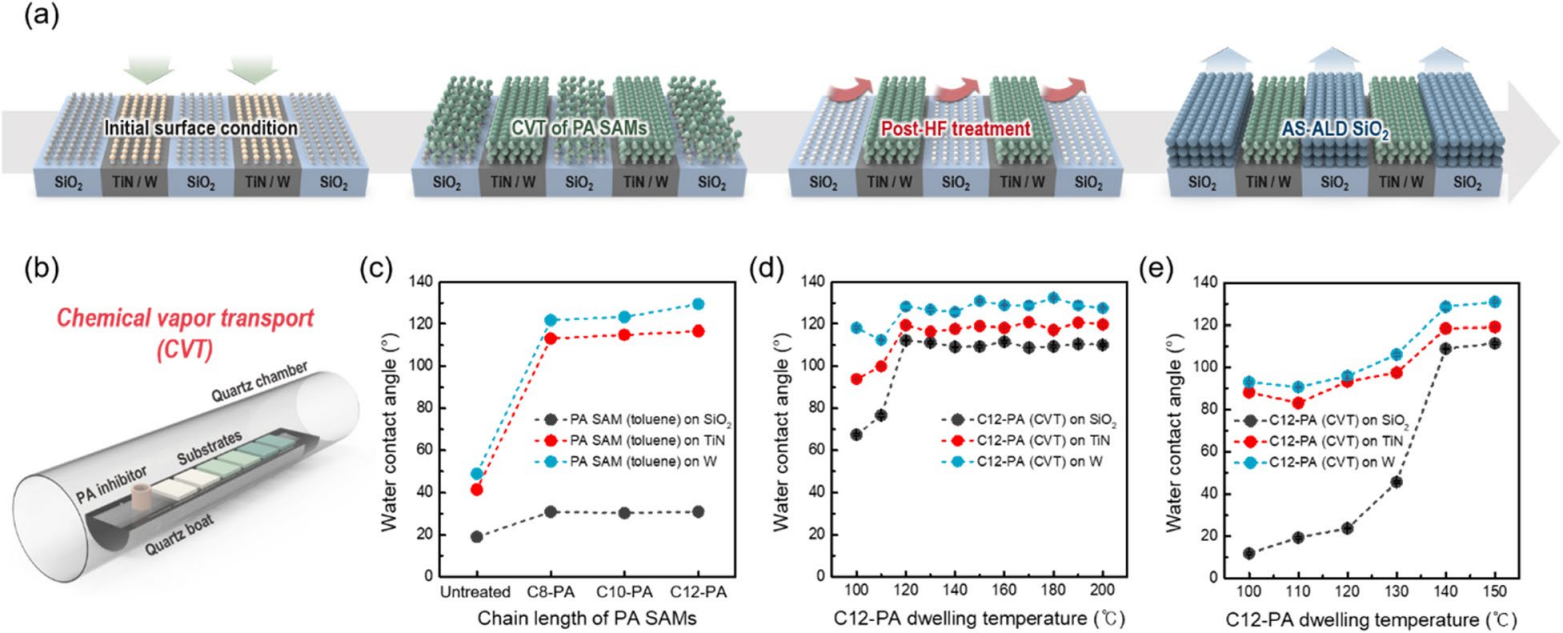
**a**, **b** Schematic illustration of (**a**) AS-ALD of SiO_2_ thin films on SiO_2_ (growth area) versus TiN and W (non-growth area) surfaces and **b** chemical vapor transport (CVT) process. **c** Water contact angle (WCA) values of SiO_2_, TiN, and W substrates after PA SAMs solution treatment with toluene solvent after 24 h of dipping at RT. **d**, **e** WCA values after C12-PA vapor dosing on SiO_2_, TiN, and W substrates as a function of substrate temperature with (**d**) 10 min and (**e**) 1 min of C12-PA dwelling time

Figure 1a illustrates the implementation of AS-ALD for SiO_2_ thin films on dielectric (i.e., SiO_2_) versus metal (i.e., TiN, W) surfaces. The goal of this study is to prevent the film growth on TiN and W substrates, ensuring that ALD SiO_2_ films were deposited exclusively on SiO_2_ substates. For this purpose, we used phosphonic acid (PA) inhibitors, denoted as ‘C8-PA, C10-PA, and C12-PA’ according to the number of carbon chains (see Figure S1), which are known to adsorb on metal and metal oxide surfaces while generally exhibiting limited reactivity with SiO_2_ substrates [21, 36]. Since the PA inhibitors used in this study are in powder form, which has an insufficient pressure for its own volatilization, a chemical vapor transport (CVT) process was introduced for facile adsorption of solid inhibitor molecules [31]. Herein, the schematic diagram of the CVT method is presented in Fig. 1b. The CVT process involves heating the chamber to efficiently expedite the chemisorption of long-chain SAM, though this process also poses a risk of unintended adsorption of PA SAMs on SiO_2_ surface. To address this, a post-HF treatment was conducted to selectively remove PA inhibitors adsorbed on SiO_2_, thereby enabling selective deposition of ALD SiO_2_ thin films on SiO_2_ substrates.

First of all, we aimed to develop an optimized CVT process with water contact angle (WCA) values comparable to those obtained via the solution process. Therefore, as a control experiment to assess the surface hydrophobicity of the PA-treated substrate, a solution treatment of PA inhibitor was performed for 24 h as shown in Fig. 1c. When PA inhibitors chemisorbed onto substrates, hydrophobic fluorocarbon moieties remain on the surfaces, leading to an increase in WCA values. Figure 1c shows the WCA variation of SiO_2_, TiN, and W substrates before and after immersion in C8-PA, C10-PA, and C12-PA solutions in toluene solvent. The chemoselective adsorption of PA inhibitors on TiN and W surfaces was confirmed by the increase in WCA value, with the extent of the increase being proportional to the length of the carbon chain. Distinct from the above cases, no notable increase in the WCA values was found on SiO_2_ substrates regardless of the carbon chain lengths, which is primarily due to the limited chemical reactivity of PA inhibitors with SiO_2_ at room temperature (RT) where the solution process was performed.

As for development of CVT process, we initially assessed substrate temperature-dependent adsorption by measuring WCA values at dwelling times of 10 min and 1 min, respectively. These CVT experiments primarily focused on C12-PA inhibitors, as they exhibited the highest WCA values in the solution-based process. In comparing Fig. 1d, e with the results in Fig. 1c, it appears that C12-PA inhibitors began to chemisorb at temperatures above 120 ℃ with dwelling time of 10 min, and above 140 ℃ with dwelling time of 1 min. Notably, the WCA values for SiO_2_ substrates also increased following the C12-PA CVT process, indicating that chemisorption of C12-PA occurred on SiO_2_ surfaces as well. These results suggest that effect surface modification with an inhibitor can be achieved in as short as 1 min, a significant improvement over the solution process, which typically requires over 24 h.

**Fig. 2:**
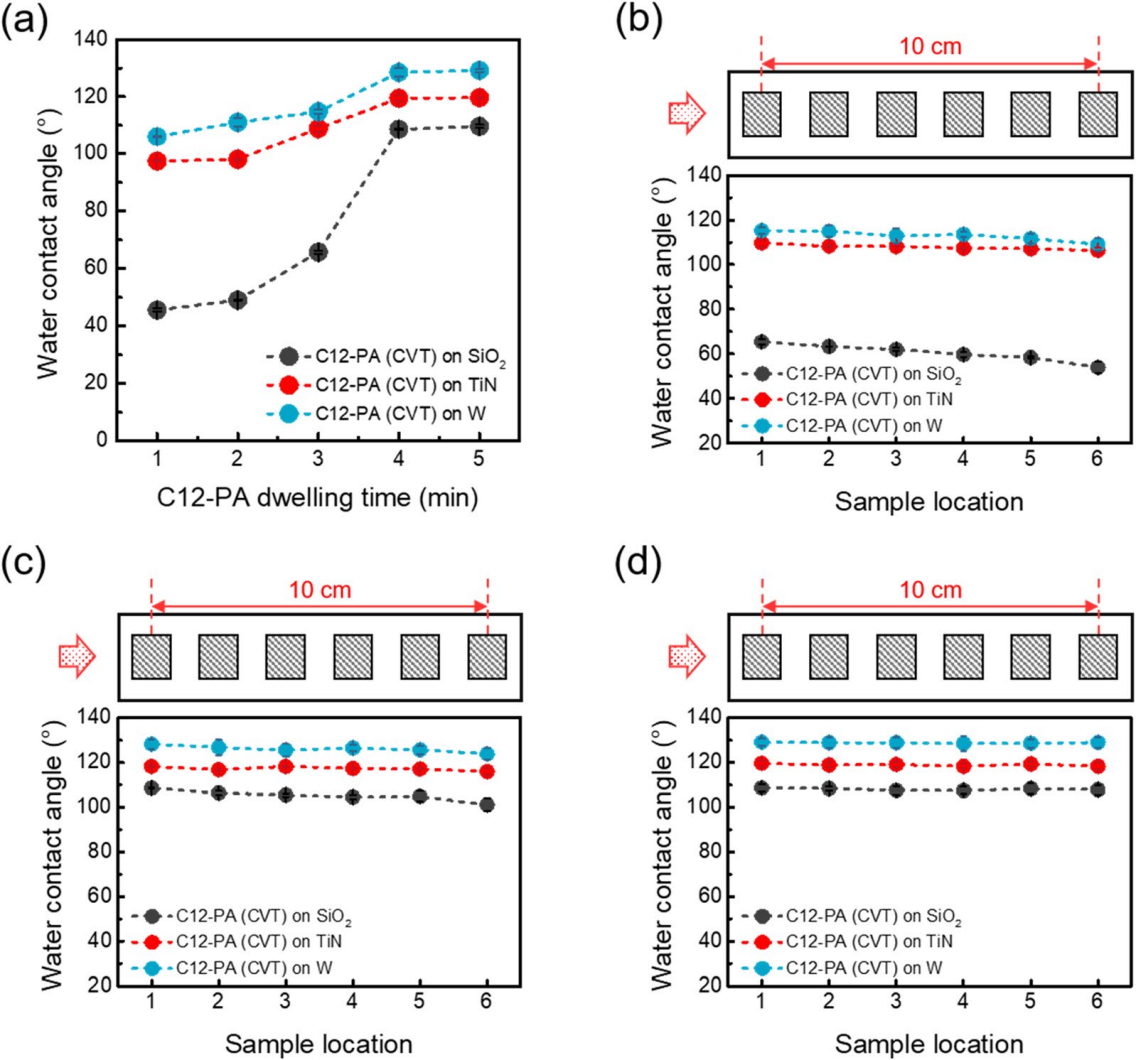
**a** WCA values after C12-PA vapor dosing on SiO_2_, TiN, and W substrates as a function of C12-PA dwelling time with substrate temperature of 130 ℃. **b**–**d** WCA values for C12-PA treated SiO_2_, TiN, and W substrates at 130 ℃ for different locations in the reactor with **b** 3 min, **c** 5 min, and **d** 10 min of dwelling time

Next, we investigated the adsorption changes of C12-PA inhibitors over different process times at a substrate temperature of 130 ℃, where the WCA values begin to show noticeable changes, as illustrated in Fig. 1e, and evaluated the uniformity across the sample position. Figure 2a shows the WCA results as a function of the dwelling time, revealing that the C12-PA inhibitors readily chemisorbed onto SiO_2_, TiN, and W substrates within just a few minutes of CVT-exposure. After dwelling times longer than 4 min, the WCA values reached saturation at approximately 128° on W substrates, 120° on TiN substrates, and 110° on SiO_2_ substrates. Figures 2b–d illustrate the adsorption variation across six samples positioned along a 10 cm length on the quartz boat within the hot-wall reactor after dwelling times of 3, 5, and 10 min, respectively, at the same temperature of 130 ℃. Note that the red arrow marks the position of the crucible containing the C12-PA inhibitors. When the C12-PA inhibitor was given insufficient time to vaporize (i.e., at a dwelling time of 3 min), a relatively high WCA value was observed at position 1 close to the crucible. At dwelling times exceeding 5 min, the WCA values reached saturation across nearly all sample positions, and a fairly uniform distribution of WCA values was confirmed after 10 min of dwelling time. These results indicate that sufficient dwelling time beyond achieving saturation in WCA value is required to ensure uniform inhibitor coating regardless of sample location.

**Fig. 3:**
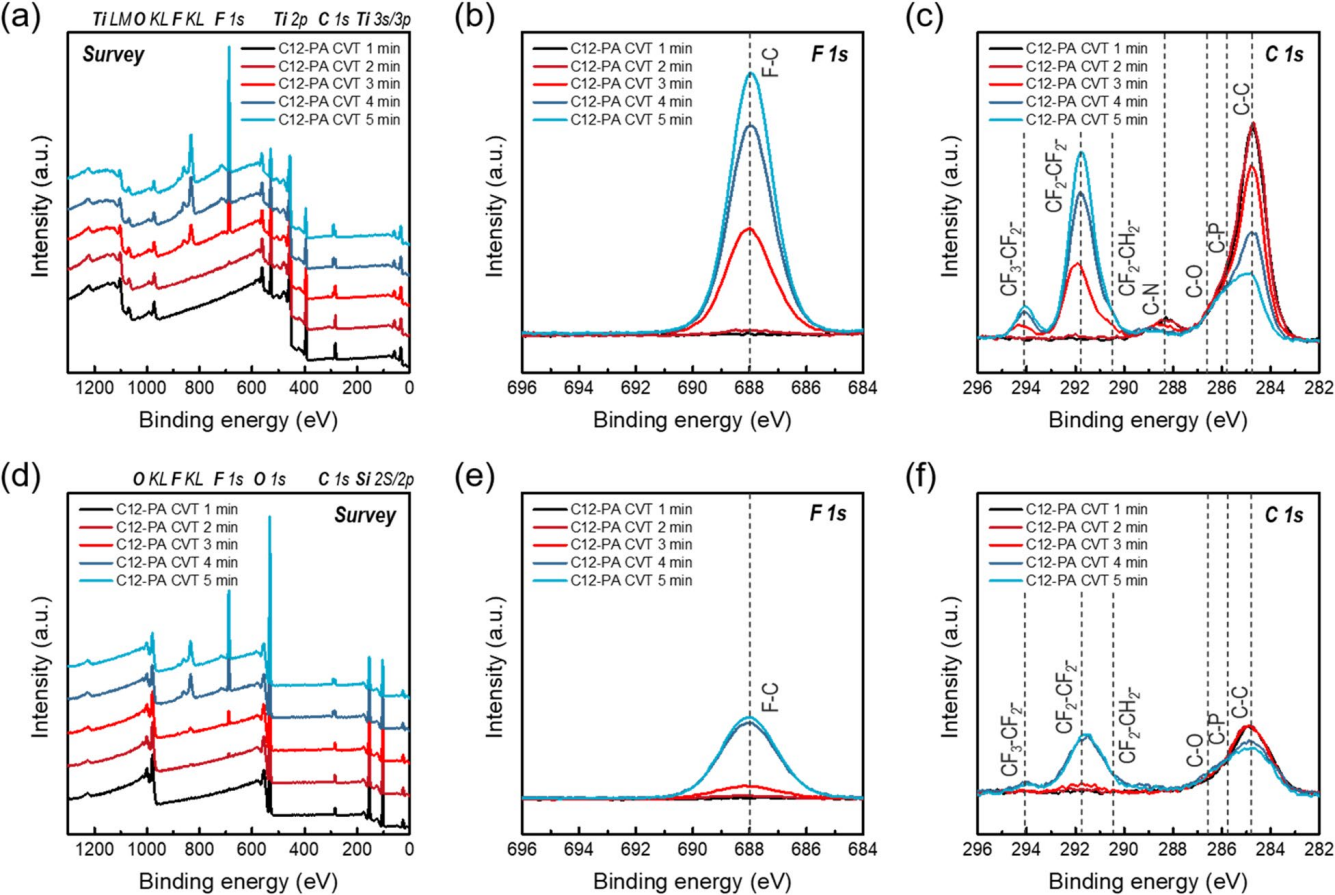
**a** XPS survey scan spectra, **b** F 1 s and **c** C 1 s core-level XPS spectra collected after C12-PA vapor dosing on TiN substrates with dwelling times of 1–5 min at 130 ℃. **d** XPS survey scan spectra, **e** F 1 s and **f** C 1 s core-level XPS spectra collected after C12-PA vapor dosing on SiO_2_ substrates with dwelling times of 1–5 min at 130 ℃

To investigate the changes in chemical properties of the C12-PA treated substrates, particularly TiN and SiO_2_, XPS analyses were conducted as a function of CVT dwelling times, with process conditions aligned to those used in Fig. 2a. The XPS survey scan spectra in Fig. 3a and d reveal a noticeable increase in F signal intensity as dwelling time increases, suggesting the progressive chemisorption of C12-PA inhibitors on TiN and SiO_2_ substrates, respectively. The F 1 s core-level XPS spectra, shown in Fig. 3b and e, illustrate a single symmetric peak centered at 688 eV, indicating F–C bonding associated with the fluorocarbon moieties of the C12-PA inhibitors [22]. Deconvolution of the C 1 s spectrum in Figure S2 shows that TiN surfaces treated with C12-PA after dwelling times of 3, 4, and 5 min were composed of C–C (284.8 eV), C-P (285.8 eV), C-O (286.6 eV), C-N (288.5–288.9 eV), CF_2_-CH_2_- (290.5 eV), CF_2_-CF_2_ (291.8 eV), and CF_3_-CF_2_- (294.1 eV), which are in good agreement with previous literature [37, 38]. To obtain an overview of the changes in the chemical bonding properties over varying dwelling times, the C 1 s core-level XPS spectra for TiN and SiO_2_ substrates were collected as shown in Fig. 3c and f, respectively. In alignment with the WCA results in Fig. 2a, the fluorine component started to increase after 3 min of dwelling time, while most of the C–C bonding attributed to adventitious carbon was gradually replaced by CF_3_- and CF_2_- moieties due to the chemisorption of the C12-PA inhibitors. For quantitative comparison depending on the dwelling time, the XPS compositional results of TiN and SiO_2_ substrates following C12-PA vapor dosing with dwelling times of 1–5 min are summarized in Tables 1 and 2, respectively. These results revealed that, unlike the WCA results, which reach saturation after a dwelling time of 4 min, the fluorine and phosphorus components continue to increase without saturation as the dwelling time is extended, again suggesting that sufficient dwelling time more than reaching saturation in WCA values is essential to achieve a densely packed inhibitor layer through the CVT process. This discrepancy in saturation behavior highlights the complementary nature of WCA and XPS measurements; WCA provides insights into macroscopic surface properties, while XPS offers detailed chemical information at the molecular scale.

**Table 1:** Summary of XPS compositional results after C12-PA vapor dosing on TiN substrates with dwelling times of 1–5 min at 130 ℃

Compositional analyses	Dwelling time	Ti 2p	N 1 s	F 1 s	C 1 s	P 2p	O 1 s
C12-PA on TiN	1 min	29.60	28.09	0.78	22.24	0.03	19.26
	2 min	29.03	27.68	1.41	22.80	0.05	19.03
	3 min	22.49	22.26	18.4	20.56	0.53	15.75
	4 min	18.41	18.28	30.36	19.45	0.88	12.64
	5 min	17.08	16.46	34.72	18.71	0.96	12.06

**Table 2:** Summary of XPS compositional results after C12-PA vapor dosing on SiO_2_ substrates with dwelling times of 1–5 min at 130 ℃

Compositional analyses	Dwelling time	Si 2p	F 1 s	C 1 s	P 2p	O 1 s
C12-PA on SiO_2_	1 min	32.29	0.24	5.37	0	62.11
	2 min	32.55	0.63	5.59	0	61.23
	3 min	31.86	2.26	6.16	0.04	59.68
	4 min	27.66	10.87	9.23	0.3	51.94
	5 min	27.49	11.77	9.19	0.36	51.18

Having clarified the surface modification by PA inhibitors via the CVT process, we assessed their blocking performance against subsequent SiO_2_ ALD process using BDIPADS precursor and O_3_ reactant on untreated and C12-PA inhibitor-treated SiO_2_ substrates. Note that the difference in the resulting SiO_2_ thickness served as an indicator of the blocking effectiveness of inhibitors, and the dwelling process was conducted under two conditions, 10 min and 3 h, based on previous findings indicating that sufficient process time was required. Given the high oxidizing power of the O_3_ reactant used in this experiment, which can readily damage the inhibitor, we examined the stability of C12-PA inhibitor by exposing it to O_3_ reactant at substrate temperatures ranging from 50 to 250 ℃ prior to film deposition. Figure S3 shows changes in the WCA values of C12-PA treated SiO_2_, TiN, and W substrates following O_3_ exposure for 900 s, plotted as a function of substrate temperature. Upon O_3_ exposure at temperatures above 200 ℃, the WCA values of C12-PA treated substrates decreased gradually as surface C12-PA inhibitor layers reacted with strong oxidizing agent. We confirmed that the WCA values of the substrates coated for 10 min decreased more significantly than those coated for 3 h, and the highest decrease in WCA was observed on the W substrate, suggesting that it may be most vulnerable to O_3_ exposure.

**Fig. 4:**
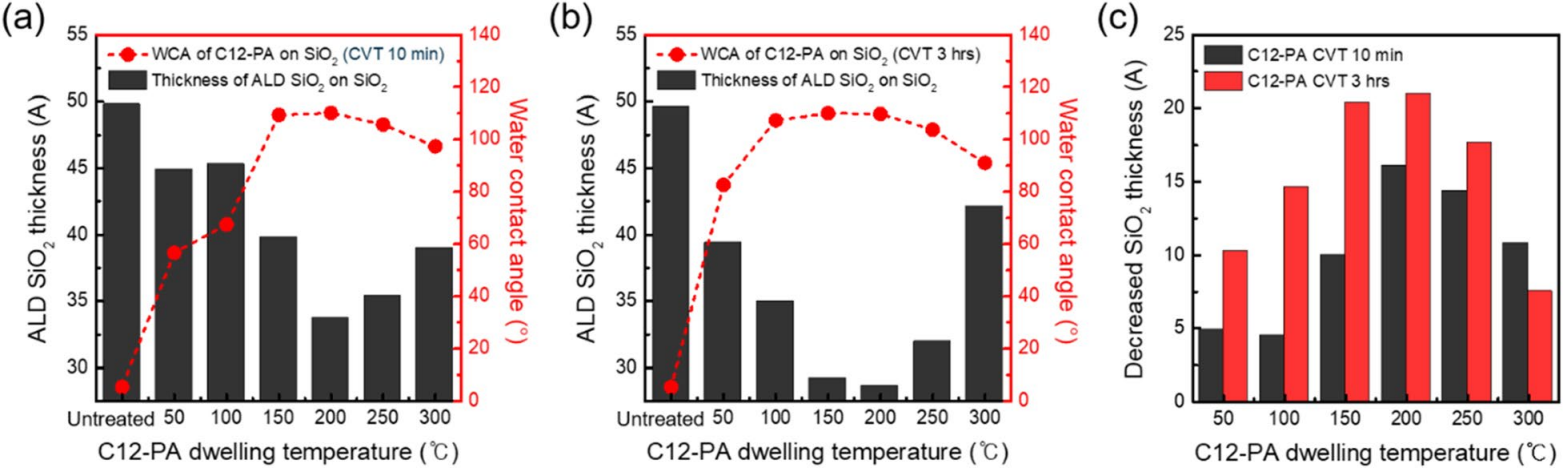
**a**, **b** WCA values before and after C12-PA vapor dosing on SiO_2_ substrates with dwelling time of (**a**) 10 min and (**b**) 3 h (red). Thickness variation of SiO_2_ thin films on untreated and C12-PA treated SiO_2_ substrates as a function of C12-PA dwelling temperature (black). **c** Blocking capability of C12-PA treated for 3 h compared to that with 10 min plotted as a function of the dwelling temperature

ALD blocking tests were then conducted at a substrate temperature of 150 ℃, a temperature at which inhibitor degradation does not occur. Figures 4a, b show the thickness variation of SiO_2_ thin films deposited on untreated and C12-PA treated SiO_2_ substrates after 50 ALD cycles, measured by spectroscopic ellipsometry, as a function of CVT dwelling temperature for the C12-PA inhibitor. The results indicate that the blocking effect aligns with the WCA trend, that is, the higher WCA values lead to greater suppression of SiO_2_ film deposition. Moreover, even when WCA values reached the same saturation point, samples with extended CVT dwelling times exhibited superior inhibitory efficacy. In the current experimental results, the largest difference in the SiO_2_ thickness was determined to be 1.6 nm for a 10 min dwelling time and 2.1 nm for a 3 h dwelling time at the same dwelling temperature of 200 ℃, as shown in Fig. 4c. Afterwards, the blocking capability of C12-PA diminishes as the CVT process temperature rises, suggesting a gradual thermal decomposition of the inhibitor, with a notable reduction observed at 300 ℃. This aligns with previous literature reporting that the C12-PA inhibitor thermally decomposes around 570–575 K [39]. Consequently, the CVT process conditions were optimized with a dwell time of 3 h at a substrate temperature of 200 ℃ to maximize the blocking capability of C12-PA inhibitors.

**Fig. 5:**
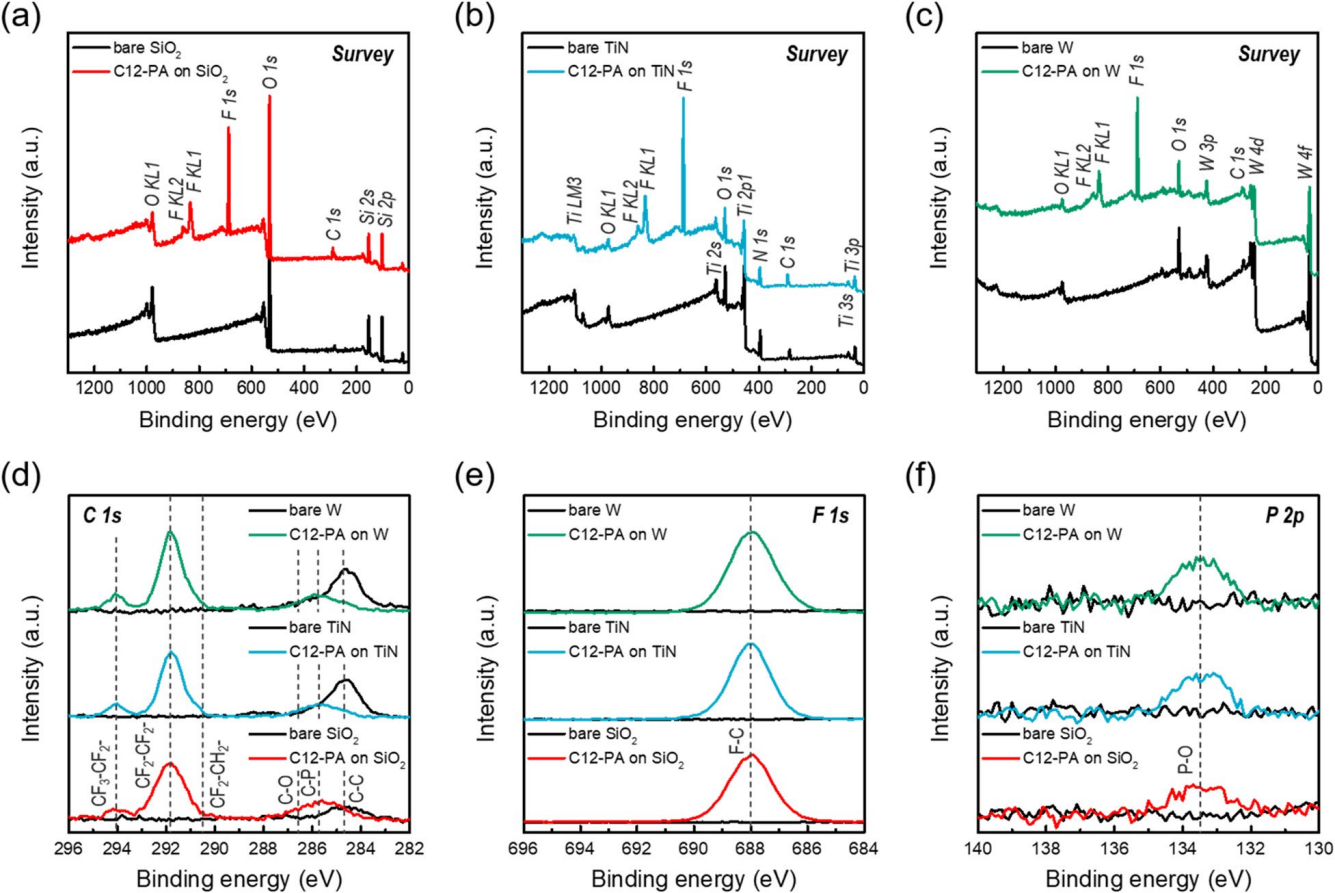
**a**–**c** XPS survey scan spectra before and after C12-PA vapor dosing on **a** SiO_2_, **b** TiN, and **c** W substrates. **d** C 1 s, **e** F 1 s, and **f** P 2p core-level XPS spectra of C12-PA treated SiO_2_, TiN, and W substrates with dwelling time of 3 h at 200 ℃

Additionally, we further investigated changes in chemical composition and bonding structure of samples treated under optimized CVT process conditions. Figure 5a–c present the XPS survey scans over a binding energy range of 0–1300 eV for untreated (denoted as ‘bare’) and C12-PA treated SiO_2_, TiN, and W substrates. Upon C12-PA treatment, all three samples exhibited a significant increase in intensity of fluorine components (F 1 s, KL1, and KL2), and the C 1 s intensity was also slightly higher than that of the bare control sample. Figure 5d–f show the detailed spectra for C 1 s, F 1 s, and P 2p region for bare and C12-PA treated substrates, revealing distinct surface bonding, including CF_2_-CF_2_ (291.8 eV), CF_3_-CF_2_ (294.1 eV), F–C (688 eV), and P-O (133.5 eV), after 3 h of dwelling time at 200 ℃. Table 3 presents the XPS-determined atomic concentration (%) for C12-PA treated SiO_2_, TiN, and W substrates. Compared with Tables 1 and 2, these results indicate that an extended dwelling time and higher substrate temperature can enhanced the chemisorption of C12-PA inhibitors on each substrate surface. Notably, the fluorine content of the C12-PA inhibitor formed under optimized CVT process conditions was approximately twice as high on the TiN and W substrate surfaces compared to the SiO_2_ substrate.

**Table 3:** Summary of XPS compositional results after C12-PA vapor dosing on SiO_2_, TiN, and W substrates with dwelling times of 3 h at 200 ℃

Compositional analyses	Si 2p	Ti 2p	N 1 s	W 4f	O 1 s	C 1 s	P 2p	F 1 s
C12-PA SiO_2_	23.44				42.42	10.49	0.57	23.08
C12-PA TiN		14.07	11.53		12.59	18.23	1.07	42.5
C12-PA W				14.48	16.94	24.01	1.6	42.98

**Fig. 6:**
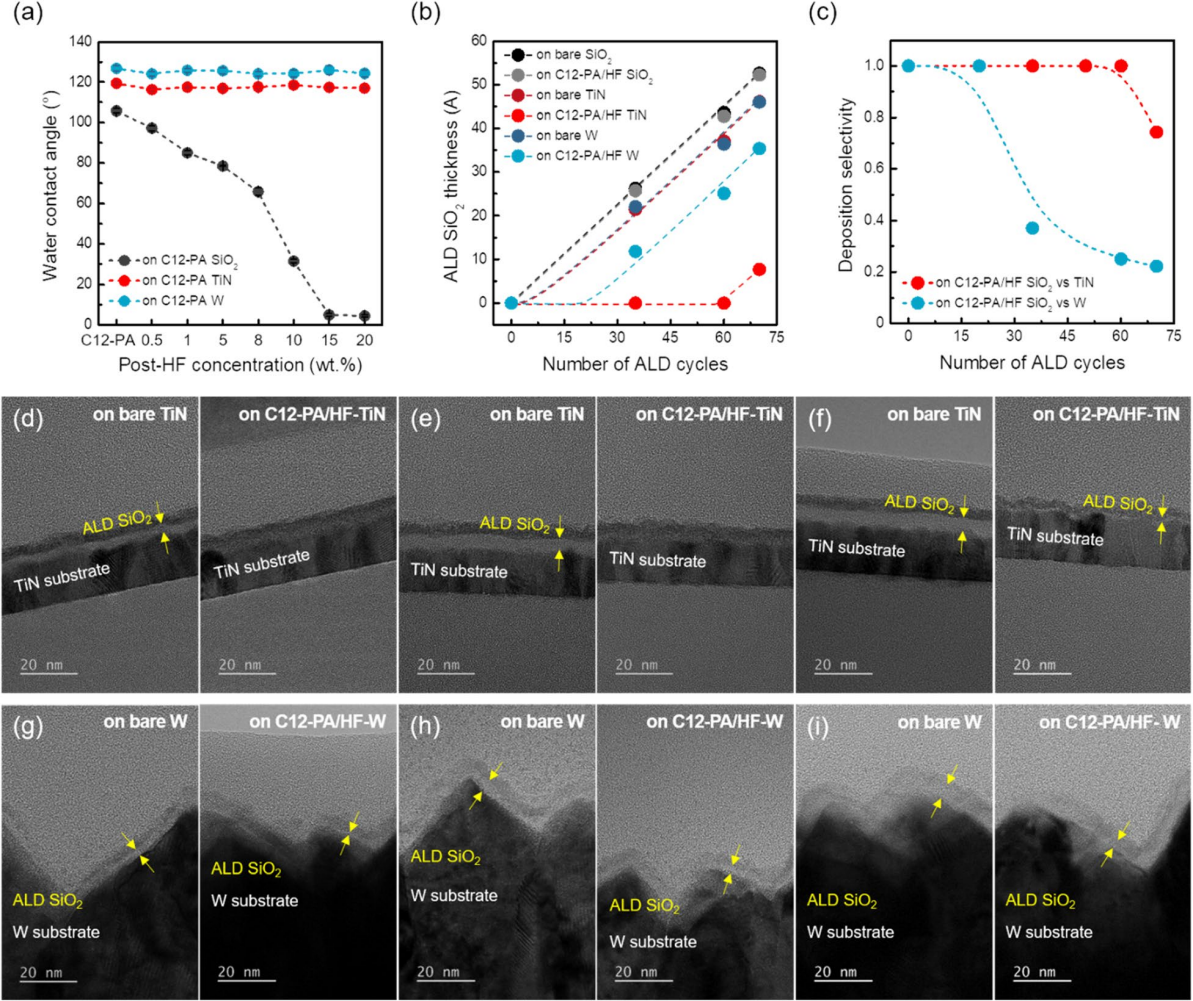
**a** WCA values of C12-PA-treated SiO_2_, TiN, and W substrates after wet-HF treatment as a function of HF concentration with treatment time of 1 s. **b** Thickness of ALD SiO_2_ thin films plotted as a function of the number of ALD cycles on untreated and C12-PA/HF treated SiO_2_, TiN, and W substrates. **c** Deposition selectivity plotted as a function of the number of ALD cycles. **d**–**f** TEM images of untreated and C12-PA/HF-treated TiN substrate after **d** 35 cycles, **e** 60 cycles, and **f** 70 cycles of SiO_2_ ALD at 100 ℃. **g**–**i** TEM images of untreated and C12-PA/HF-treated W substrate after **g** 35 cycles, **h** 60 cycles, and **i** 70 cycles of SiO_2_ ALD at 100 ℃

Finally, to achieve selective deposition between SiO_2_ and metal surfaces, we introduced an ex-situ post-HF treatment following the CVT process, as illustrated in Fig. 6a. This treatment selectively removes the PA inhibitor from SiO_2_ surfaces, consistent with findings from our previous work [31]. WCA measurements taken before and after the post-HF treatment under various HF concentrations revealed that only the WCA of C12-PA treated SiO_2_ substrates decreased selectively with increasing HF concentration, while the TiN and W substrates remained unaffected. These results suggest that the interfacial SiO layer covered by C12-PA inhibitors is selectively removed after HF treatment, leaving surface -OH functional groups on SiO_2_ substrates. This functionalization promotes selective SiO_2_ film deposition exclusively on SiO_2_ surfaces, while metal surfaces remain passivated with -CF_3_ functional groups during the initial stages of the ALD process. The selective etching results can be attributed to the following factors: (i) HF alone does not etch TiN or W surfaces, as oxidation or chemical conversion must precede to enable etching of these interfacial layers with HF [40, 41]. (ii) The Si–O-P bond on SiO_2_ surface is sensitive to hydrolysis, unlike the metal-O-P bonds, making it readily removable through HF wet treatment followed by DIW rinsing [42]. Further P 2p core-level XPS spectra were provided to clarify the selective removal of C12-PA inhibitors from SiO_2_ substrates, as shown in Figure S4. Therefore, we applied HF-wet etching (10 wt%, 1 s) after the CVT process to selectively remove the C12-PA inhibitor from SiO_2_ surfaces. Notably, these mild HF conditions did not affect the thickness or morphology of the TiN and W substrates, as shown in Figure S5.

Subsequent SiO_2_ ALD process was then proceeded at a substrate temperature of 100 ℃ to minimize degradation of the C12-PA inhibitor. Figure 6b show the thickness variation of ALD SiO_2_ films on both untreated and C12-PA/HF-treated SiO_2_, TiN, and W substrates with increasing number of ALD cycles to determine where the selectivity loss occurs. The thickness of the ALD SiO_2_ films was cross-checked by combining spectroscopic ellipsometry and transmission electron microscopy (TEM) to ensure measurement accuracy. Figure S6 displays the thickness of untreated and C12-PA/HF-treated SiO_2_ substrates measured by an ellipsometer as a SiO_2_ model at each process step: before ALD process, after CVT process, after post-HF treatment, and after 35, 60, and 75 cycles of the ALD process. Note that the slight increase in thickness observed after the CVT process is attributed to the chemisorption of C12-PA inhibitor, and the SiO_2_ substrates decreased to the same thickness as the bare state after HF treatment. As illustrated in Fig. 6b, after C12-PA exposure combined with the post-HF treatment, negligible variation in SiO_2_ thickness between untreated and treated SiO_2_ substrates, consistent with the WCA results in Fig. 6a, indicating selective removal of C12-PA inhibitors by HF treatment. Conversely, a significant distinction was observed on metal surfaces, particularly for TiN substrates, following the C12-PA inhibitor and post-HF treatment, attributable to the chemisorbed PA inhibitor molecules. The C12-PA inhibitors significantly retarded the growth of SiO_2_ during the ALD process, suppressing SiO_2_ film deposition by up to 1 nm on W substrates and up to 4 nm on TiN substrates. Furthermore, the practical definition of deposition selectivity (S) in AS-ALD process can be typically expressed as S = (*θ*_G_ – *θ*_NG_)/(*θ*_G_ + *θ*_NG_), where *θ*_G_ and *θ*_NG_ indicate the amount of material deposited on the growth area (*i.e.*, SiO_2_ surface) and non-growth area (*i.e*., TiN and W surfaces). Based on this definition, Fig. 6c shows the calculated selectivity of ALD SiO_2_ thin films on SiO_2_ versus TiN and W surfaces, plotted versus ALD cycle number. The results indicate that S = 1 can be maintained up to 20 cycles on W substrates and up to 60 cycles on TiN substrates, compared to SiO_2_ substrates. Figure 6d–i and S7 present cross-sectional TEM images of ALD SiO_2_ films deposited on SiO_2_, TiN and W substrates, corresponding to the data shown in Fig. 6b, while Figure S8 provides additional images taken at different magnifications.

Interestingly, our findings revealed that while the W surface exhibited high WCA values following C12-PA inhibitor treatment, its ALD blocking performance was notably poorer than that of TiN surfaces. This result contrasts with a previous study that ODPA-treated W substrate demonstrated excellent blocking properties, suggesting that the characteristics of the underlying substrate significantly influence the formation of high-quality SAMs. Regarding this, Bobb-Semple et al. reported that substrate surface roughness critically affects SAM stability [21, 43]. Their study compared the adsorption and blocking capabilities of ODPA SAMs on Co, Cu, W, and Ru substrates, and the results showed that (i) ODPA-treated W substrate achieved the greatest suppression of ZnO and Al_2_O_3_ film deposition among other substrates, and (ii) a disordered ODPA layer with randomly oriented chains formed on Ru surfaces with high surface roughness. As evidenced by the SEM and TEM images in Figures S4 and 6, the W substrate used in our study exhibited high surface roughness, which likely led to the formation of an unstable inhibitor layer. This instability may result in a relatively low blocking effect for subsequent ALD process and make the W substrate particularly vulnerable to O_3_ exposure, as shown in Figure S3. These findings highlight that substrate characteristics, including surface roughness, are pivotal to achieving an inhibitor-compatible AS-ALD process. Therefore, careful consideration of substrate preparation is also crucial to ensure effective blocking performance and enable the formation of high-quality SAM inhibitors.

## Conclusions

We have demonstrated a robust strategy for achieving AS-ALD of SiO_2_ thin films on SiO_2_ versus metal surfaces by leveraging vapor-dosed bulky SAMs combined with the selective removal of C12-PA from SiO_2_ substrates. A bulky solid PA inhibitor was effectively vaporized within a short period using the CVT process, and deposition selectivity between SiO_2_ and metal surfaces was secured by introducing a post-HF treatment. It is worth noting that a superior blocking capability of vapor-dosed C12-PA SAMs was successfully confirmed with selective deposition of SiO_2_ films over 4.5 nm on C12-PA/HF-treated SiO_2_ versus TiN substrates, even under ALD conditions involving strong oxidizing O_3_ reactants. Furthermore, our findings emphasize that WCA measurements alone are insufficient to determine whether well-aligned SAMs have formed, and the properties of the underlying substrate also play an important role for the formation of high-quality SAMs. This study holds potential for AS-ALD applications to fabricate highly selective dielectrics on dielectrics (DoD) nanostructure for advanced bottom-up nanofabrication.

## Supplementary Information

Supplementary material 1.

## Data Availability

The datasets used and/or analyzed during the current study are available from the corresponding author on reasonable request.
