# Supplementary material for: Selective metal passivation by vapor-dosed phosphonic acid inhibitors for area-selective atomic layer deposition of SiO2 thin films
Source: Nano Converg. 2025 May 30;12:27. doi: 10.1186/s40580-025-00490-5 (PMC12125412; doi:10.1186/s40580-025-00490-5)
Supplement: Supplementary file 1 — Supplementary material 1. [file 40580_2025_490_MOESM1_ESM.docx]

Supporting Information

**Selective Metal Passivation by Vapor-Dosed Phosphonic Acid Inhibitors for Area-Selective Atomic Layer Deposition of SiO_2_ Thin Films**

Jeong-Min Lee,^a,†^ Seo-Hyun Lee,^a,†^ Ji Hun Lee,^b^ Junghun Kwak,^b^ Jinhee Lee,^b^ and Woo-Hee Kim ^a,*^

^a^Department of Materials Science and Chemical Engineering, BK21 FOUR ERICA-ACE Center, Hanyang University, Ansan, Gyeonggi 15588, Republic of Korea

^b^SK specialty Co., Ltd, 59-33 Gaheunggongdan-ro, Yeongju-si, Gyeongsangbuk-do 36059, Korea

*Corresponding author: Woo-Hee Kim

E-mail address: [wooheekim@hanyang.ac.kr](mailto:wooheekim@hanyang.ac.kr)

^†^These authors (J.-M. Lee and S.-H. Lee) contributed equally to this work.


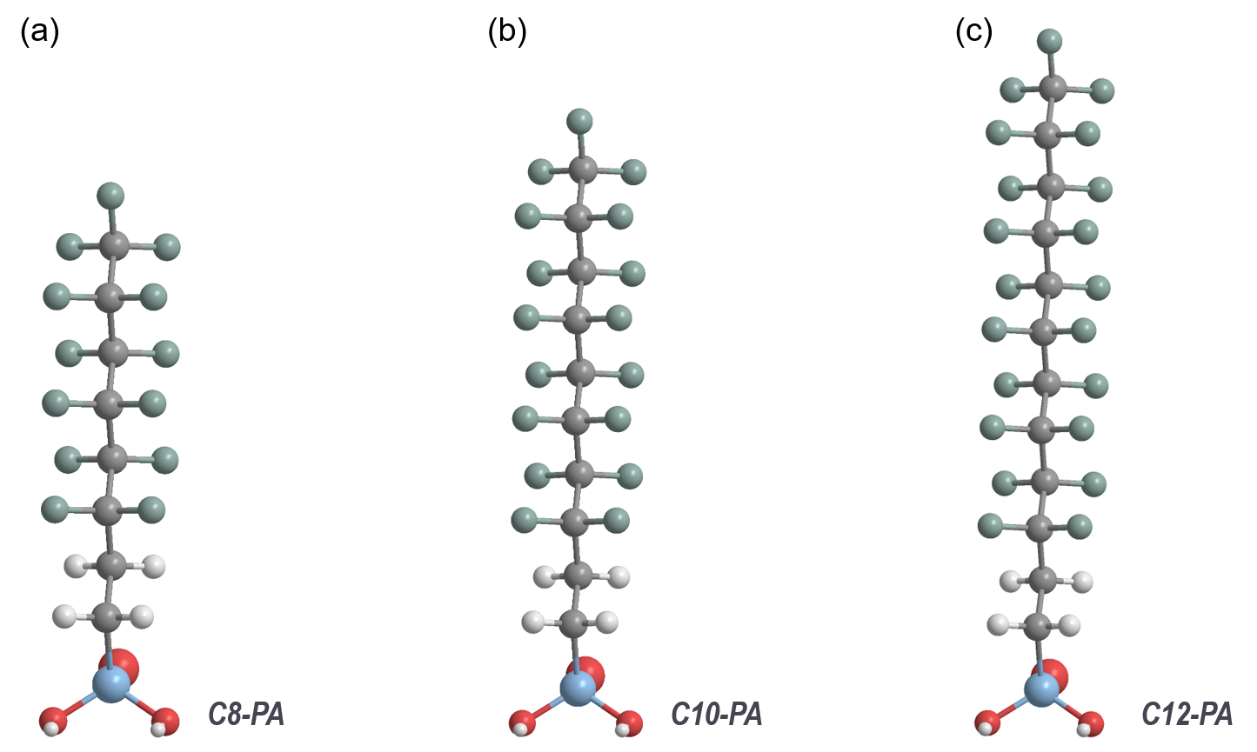


**Figure S1.** Molecular formulars of PA inhibitors used in this study: (a) C8-PA, (b) C10-PA, and (c) C12-PA molecules (blue = phosphorus, green = fluorine, red = oxygen, gray = carbon, white = hydrogen).


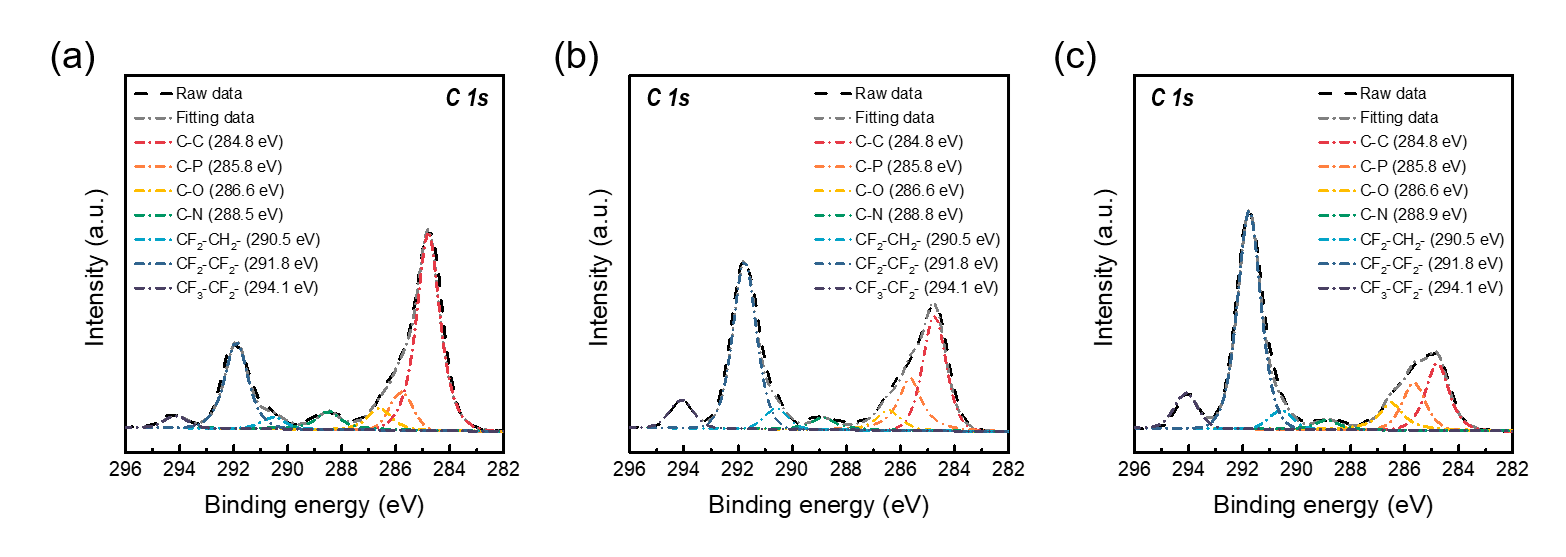


**Figure S2.** C 1s core-level XPS spectra of TiN substrates following C12-PA vapor dosing with dwelling times of (a) 3 min, (b) 4 min, and (c) 5 min at 130 ℃.


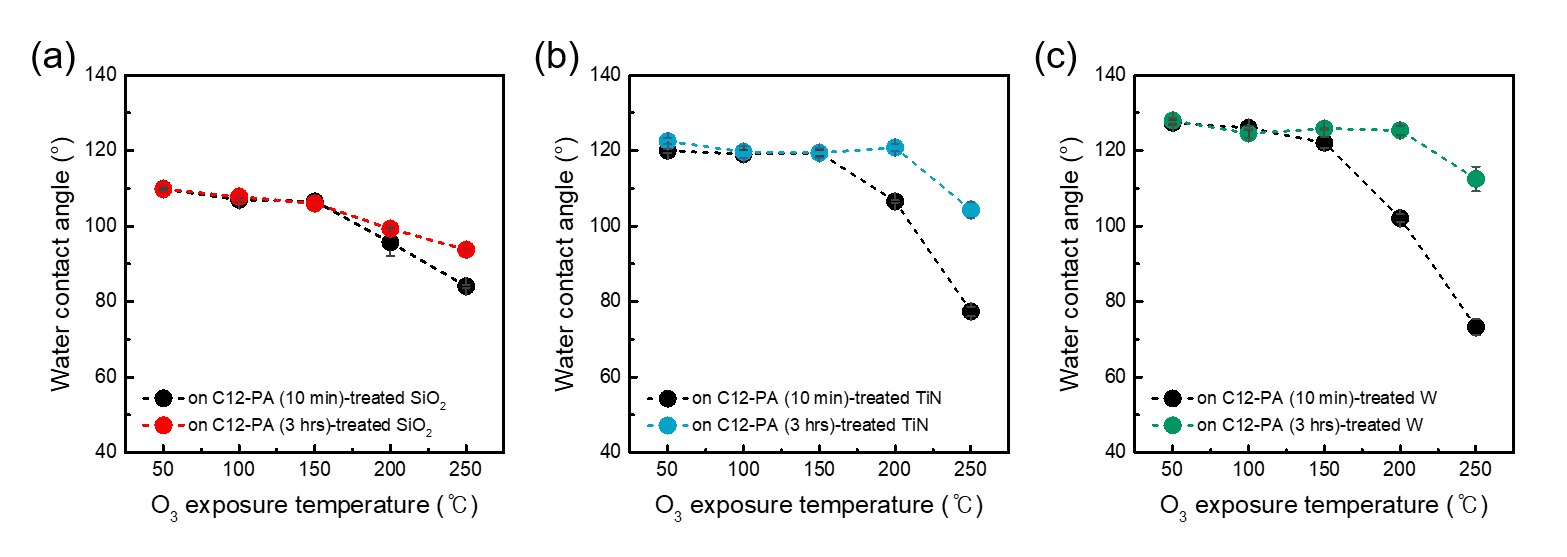


**Figure S3.** WCA values of C12-PA treated (a) SiO_2_, (b) TiN, and (c) W substrates as a function of O_3_ exposure temperature with exposure time of 900 sec.

**
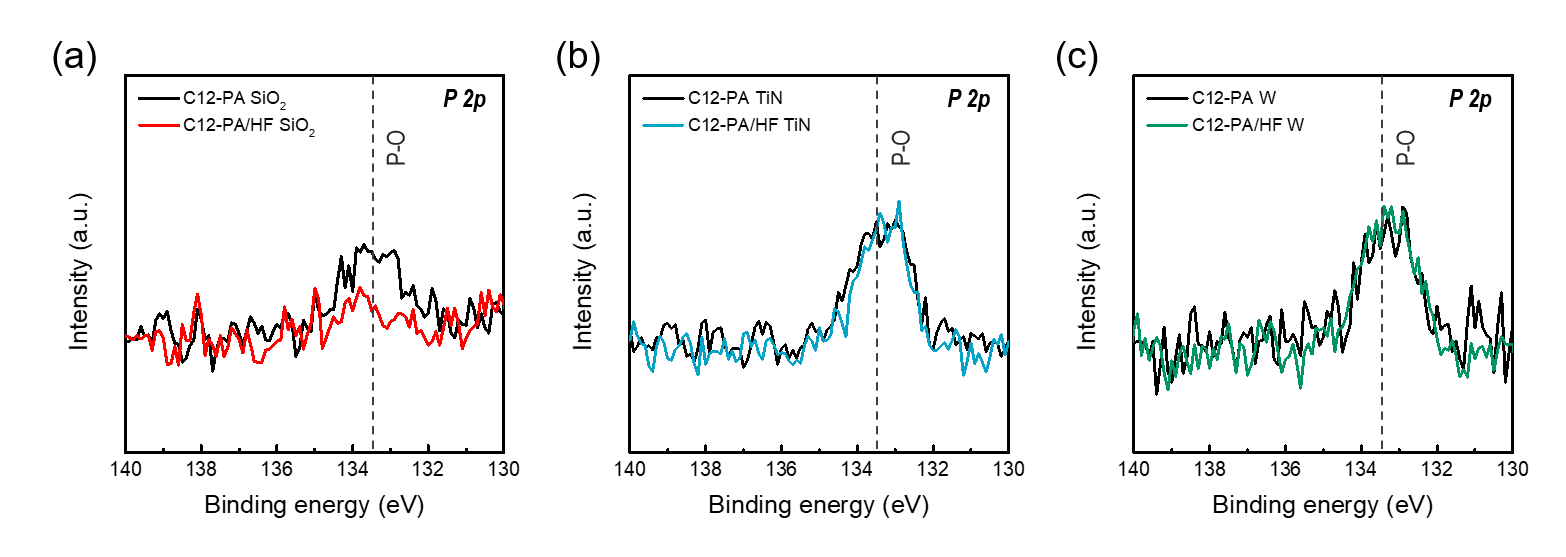
**

**Figure S4.** P 2p core-level XPS spectra before and after HF-treatment on (a) SiO_2_, (b) TiN, and (c) W substrates.


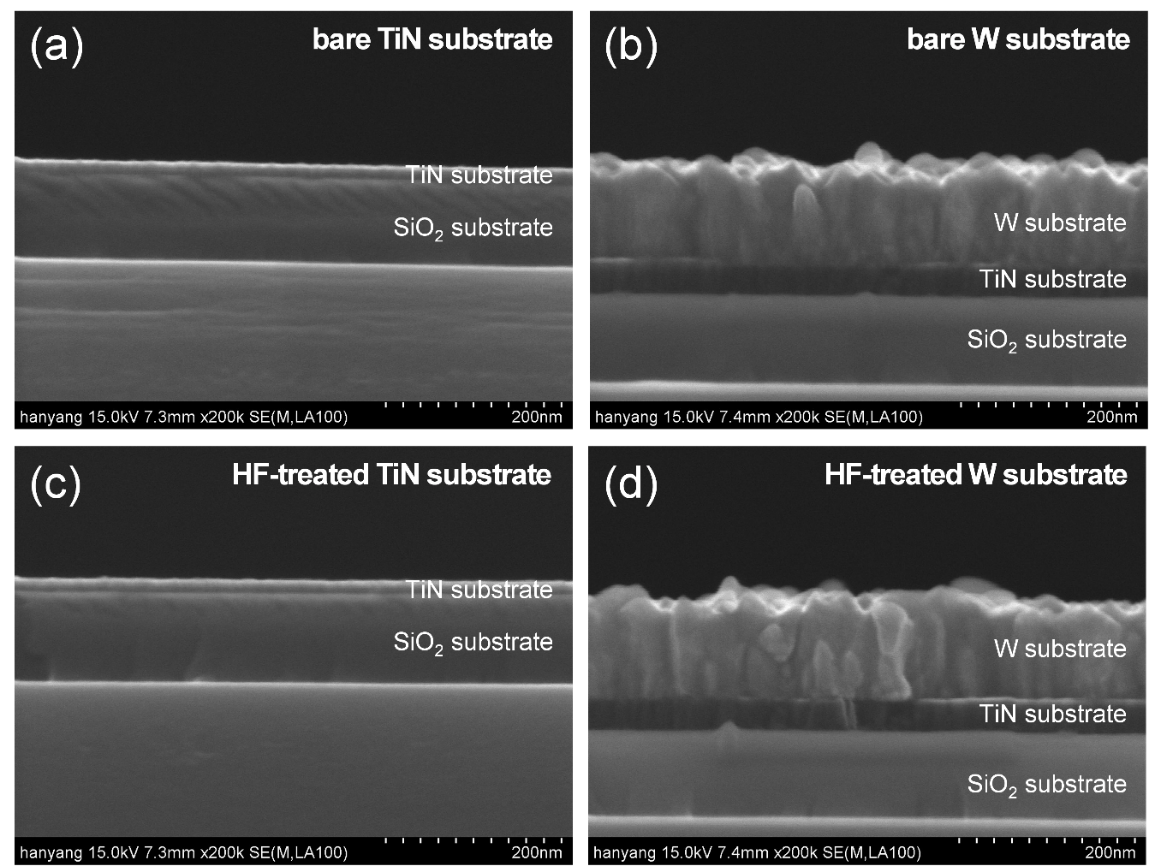


**Figure S5.** Cross-sectional SEM images of TiN and W substrates before and after HF-treatment.

**
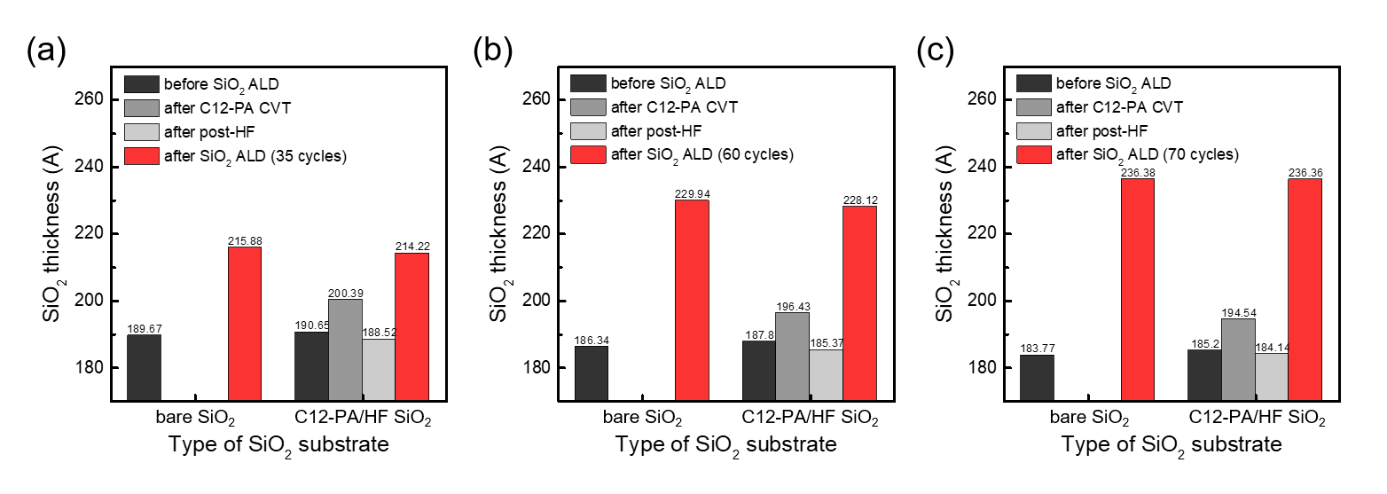
**

**Figure S6.** Thickness of SiO_2_ substrates after each process step for (a) 35 ALD cycles, (b) 60 ALD cycles, and (c) 70 ALD cycles.

**
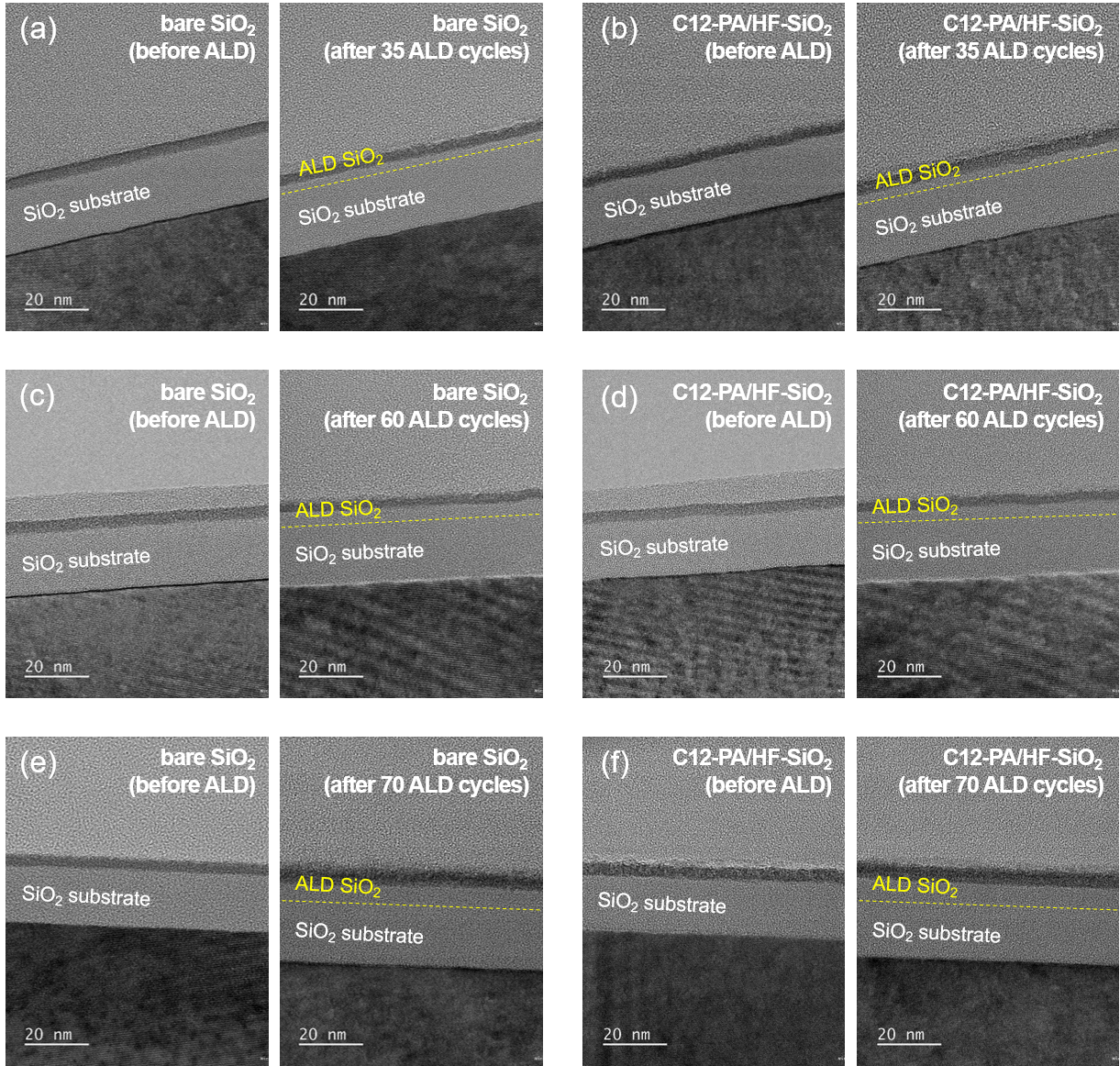
**

**Figure S7.** TEM images of untreated and C12-PA/HF-treated SiO_2_ substrates before and after (a-b) 35 cycles, (c-d) 60 cycles, and (e-f) 70 cycles of SiO_2_ ALD at 100 ℃.

**
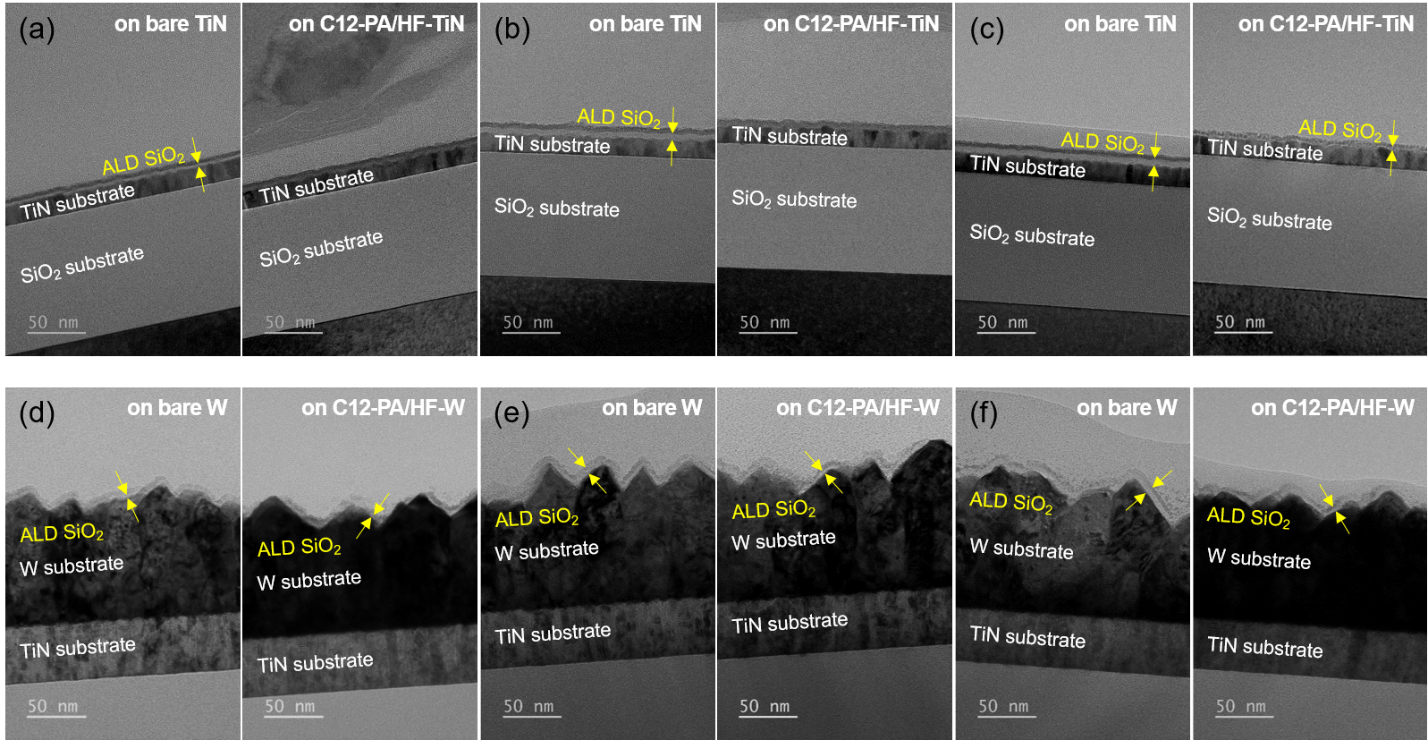
**

**Figure S8.** (a-c) TEM images of untreated and C12-PA/HF-treated TiN substrates after (a) 35 cycles, (b) 60 cycles, and (c) 70 cycles of SiO_2_ ALD at 100 ℃. (d-f) TEM images of untreated and C12-PA/HF-treated W substrates after (d) 35 cycles, (e) 60 cycles, and (f) 70 cycles of SiO_2_ ALD at 100 ℃.
